# Treatment of Urinary Tract Infections with Canephron^®^ in Germany: A Retrospective Database Analysis

**DOI:** 10.3390/antibiotics10060685

**Published:** 2021-06-08

**Authors:** Martina Höller, Hubert Steindl, Dimitri Abramov-Sommariva, Florian Wagenlehner, Kurt G. Naber, Karel Kostev

**Affiliations:** 1 Bionorica SE, Kerschensteinerstr. 11-15, 92318 Neumarkt, Germany; Martina.Hoeller@bionorica.de (M.H.); Hubert.Steindl@bionorica.de (H.S.); Dimitri.Abramov-Sommariva@bionorica.de (D.A.-S.); 2 Clinic for Urology, Pediatric Urology and Andrology, Justus-Liebig University, Giessen, Rudolf-Buchheim-Straße 7, 35392 Giessen, Germany; wagenlehner@aol.com; 3 Department of Urology, Technical University of Munich, PA: Karl-Bickleder Street 44c, 94315 Straubing, Germany; kurt@nabers.de; 4 IQVIA, Epidemiology, Unterscheinstiege 2-14, 60549 Frankfurt, Germany

**Keywords:** Canephron, antibiotic, urinary tract infections, cohort study, herbal treatment

## Abstract

*Objective:* The goal of the present study was to evaluate treatment with Canephron^®^ compared to standard antibiotic treatment after diagnosis of acute cystitis or urinary tract infection (UTI), with regard to the risk of sporadic recurrent UTIs, frequent recurrent UTIs, UTI-related sick leave, additional antibiotic prescriptions, and renal complications (pyelonephritis). *Methods:* This retrospective cohort study was based on data from the IMS^®^ Disease Analyzer database (IQVIA), and included outpatients in Germany with at least one diagnosis of acute cystitis or UTI with a prescription of either Canephron^®^ or standard antibiotics between January 2016 and June 2019 and treated in general practitioner (GP), gynecologist, or urologist practices, from which the data were obtained. Multivariable regression models were used to investigate the association between Canephron^®^ prescription and the amount of sporadic or frequent recurrent UTIs, as well as the duration of UTI-related sick leave, the number of additional antibiotic prescriptions, and cases of pyelonephritis. The effects of Canephron^®^ were adjusted for age, sex, insurance status, and Charlson comorbidity score (CCI). *Results:* 2320 Canephron^®^ patients and 158,592 antibiotic patients were available for analysis. Compared to antibiotic prescription, Canephron^®^ prescription was significantly associated with fewer sporadic recurrences of UTI infections 30–365 days after the index date (odds ratio (OR): 0.66; 95%, confidence interval (CI): 0.58–0.72), as well as less frequent recurrences of UTI infections (OR: 0.61; 95% CI: 0.49–0.88), and also with reduced additional antibiotic prescription within 31–365 days (OR: 0.57; 95% CI: 0.52–0.63). No significant differences were observed between the Canephron^®^ and antibiotic cohorts with regard to the likelihood of sick leave (OR: 0.99; 95% CI: 0.86–1.14), new antibiotic prescription within 1–30 days (OR: 1.01; 95% CI: 0.87–1.16), or occurrence of pyelonephritis (Hazard Ratio (HR): 1.00; 95% CI: 0.67–1.48). *Conclusion:* These real-world data show that Canephron^®^ is an effective, safe symptomatic treatment for acute cystitis or UTI. It should be considered as an alternative treatment, particularly to also strengthen antimicrobial stewardship strategies.

## 1. Introduction

Urinary tract infections (UTI) affect about 150 million people per year worldwide [1] and are among the leading reasons for treatment in adult primary care medicine [2]. According to Foxman, almost half of all women will experience one episode of cystitis in their lives and about one third of women will have experienced an episode of cystitis by the age of 24 [3]. Most UTIs are caused by acute uncomplicated cystitis [4] and current guidelines recommend antibiotics as the first-line therapy [5,6].

In response to the dramatic increase in the development of resistant bacteria, antibiotic stewardship calls for the cautious use of antibiotics where necessary, and recommends avoiding antibiotics where possible. As the body′s immune system is able to deal with pathogenic bacteria in most uncomplicated infections, antibiotics are not necessarily indicated in all cases of infection [7]. Accordingly, the “EU Guidelines for the prudent use of antimicrobials in human health” (2017) generally recommend that prescribers avoid antibacterial treatment when there is only evidence of a viral infection or a self-limiting bacterial infection. Based on this need to avoid antibiotics where possible, non-antibiotic, symptomatic treatments have become an important treatment option for patients with uncomplicated UTIs. To date, a number of studies have compared the efficacy of non-steroidal anti-inflammatory drugs (NSAIDs), e.g., ibuprofen [8,9] or diclofenac [10] to that of antibiotics. As these trials indicated sufficient efficacy, several guidelines now also recommend non-antibiotic treatment. According to the current EAU (European Association of Urology) guideline [5], as well as the German AWMF interdisciplinary S3 guideline [6], antibiotics are still considered the first-line treatment option for UTI, but non-antibiotic, symptomatic treatment should be considered in cases of acute uncomplicated cystitis with mild or moderate symptoms. The administration of herbal preparations is also an appropriate non-antibiotic, approach for UTI treatment [11,12]. One such product, approved in 31 countries for the short and long-term treatment of various urological diseases, is Canephron^®^, which contains centaury herbs (*Centaurium erythraea* Rafn, *herba*), lovage roots (*Levisticum officinale* Koch, *radix*), and rosemary leaves (*Rosmarinus officinalis* Linné, *folium*) [13]. Canephron^®^ has what are called “multi-target” properties, including spasmolytic [14], diuretic [15], anti-oxidative [16], anti-adhesive [17], anti-inflammatory, and anti-nociceptive effects [18].

The effectiveness of Canephron^®^ has been demonstrated in a number of clinical studies [13,19,20,21].

Recently, Wagenlehner et al. conducted a randomized, double-blind, phase III trial, which demonstrated that the treatment of women with acute lower uUTIs using Canephron^®^ was non-inferior to treatment with the antibiotic fosfomycin trometamol for the prevention of additional intake of antibiotics [21].

According to Haynes, the benefit–risk assessment of medicinal products should not only be made under study conditions (“can it work?”) but should also be proven under real world conditions (“does it work?”, “is it worth it?”) [22]. As a result, the goal of the present study was to prove the effectiveness of Canephron^®^ monotherapy as a symptomatic treatment for UTIs based on real world data under normal healthcare practice circumstances, and thereby to confirm clinical data from previous interventional studies. Therefore, we evaluated the number of Canephron^®^ prescriptions (either Canephron^®^ N or Canephron^®^ UNO) issued as treatment after the diagnosis of acute cystitis or UTI and the need for additional antibiotic prescriptions compared to the standard therapy with antibiotics. As the risk of recurrence, as well as complications and the duration of the disease, are common reasons for prescribing antibiotics, we also examined the effect of Canephron^®^ monotherapy on sporadic and frequent recurrent UTIs, pyelonephritis, and the duration of UTI-related sick leave.

## 2. Materials and Methods

### 2.1. Data Source

This analysis was based on data from the IMS^®^ Disease Analyzer database (DA), which contains case-based information provided by office-based physicians (both general practitioners (GPs) and specialists) in Germany. Information is available on patient demographics, drug prescriptions, concomitant medication, comorbid conditions, sick leave, and referrals to hospital. Our data analyses only considered data from those sites that have continuously delivered data to the IMS^®^DA panel in the past. IMS^®^DA contains data from more than 10 million patients, captured between 2015 and 2019. Information is provided by nearly 3000 office-based physicians, representing approximately 3% of all German practices. The sample of practices included is geographically representative for Germany, covering eight major German regions. In Germany, the sampling methods used for the selection of physicians′ practices are appropriate for obtaining a representative database of general and specialized practices [23].

German law allows the use of anonymous electronic medical records for research purposes under certain conditions. According to this legislation, it is not necessary to obtain informed consent from patients or approval from an institutional review board (IRB) for this type of observational study, which contains no directly identifiable data. As patients were only queried as aggregates and no protected health information was available for queries, no IRB approval was required for the use of this database or the completion of this study.

### 2.2. Study Population and Covariables

This retrospective cohort study includes patients with at least one diagnosis of acute cystitis (ICD-10: N30.0) or UTI (ICD-10: N39.0) between 1 January 2016 and 30 June 2019 in one of the GP, gynecologist, or urologist practices from which data were obtained. The first diagnosis documented during this period was considered the index date. Further inclusion criteria included at least 12 months of observation prior to this diagnosis, which was verified by the documentation of at least one visit to the physician in the period ≥365 days prior to the index date, and a prescription for either Canephron^®^ N or Canephron^®^ UNO or a standard antibiotic (ATC: J01) on the index date. Patients with an antibiotic prescription within 30 days prior to the index date, prescriptions of other herbal medications during the study period, or a prescription of both Canephron^®^ and any antibiotic drug together on the index date were excluded. Patients were categorized into one of two cohorts: the Canephron^®^ cohort, and the antibiotic cohort. Both cohorts were then compared with each other.

The covariables used in this study included age, sex, health insurance coverage (private or statutory), physician specialty (GP, gynecologist, urologist), and Charlson comorbidity index (CCI). The CCI is a method for categorizing patient comorbidities based on the International Classification of Diseases (ICD) and contains 19 categories. The higher the score, the more likely it is that the predicted outcome will result in mortality or higher resource use [24].

### 2.3. Statistical Analyses

We estimated the differences in the proportions of patients: (1) with sporadic UTI recurrence, defined as at least one renewed confirmed diagnosis of UTI (ICD-10: N39.0), or acute cystitis (ICD-10: N30.0) within 30–365 days from the initial diagnosis; (2) with frequent recurrent UTIs, defined according to the current EAU guideline [5] as at least three diagnoses of urinary tract infection 2–365 days after initial diagnosis or at least two diagnoses of urinary tract infection 2–184 days after initial diagnosis; (3) with UTI-related sick leave, defined as documented sick leave within one month following the diagnosis of a UTI; (4) with at least one antibiotic prescription 1–30 days after diagnosis or later (30–365 days); and (5) with an initial documentation of pyelonephritis (ICD-10: N10-12) within up to 3 years following the diagnosis of UTI.

Multivariable logistic regression models were used to investigate the association between Canephron^®^ prescription and a lower risk of sporadic UTI recurrence, frequent recurrent UTIs, UTI-related sick leave, and antibiotic prescriptions. The effects of Canephron^®^ were adjusted for age, sex, insurance status, and CCI. Multivariable regression models were also performed separately for men and women and for three age groups (≤40, 41–60, >60 years). A p-value of <0.05 was considered statistically significant.

The Kaplan–Meier method was used to estimate the differences between the Canephron^®^ cohort and the antibiotic cohort based on the percentage of patients with an initial documentation of pyelonephritis within up to 3 years following the diagnosis of a UTI. Patients with documented diagnoses of pyelonephritis prior to the index date were excluded from this analysis. A multivariable Cox regression model was used to investigate the association between Canephron^®^ prescription and the probability of pyelonephritis, adjusted for age, sex, insurance status, and CCI.

## 3. Results

### 3.1. Patient Selection and Baseline Characteristics of Study Patients

**Figure 1:**
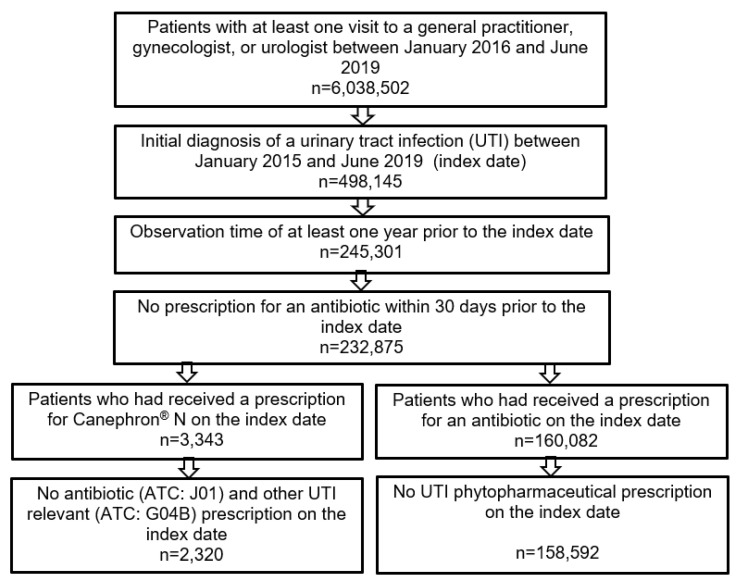
Selection of study patients.

Of the 232,875 patients diagnosed with UTI (ICD-10: N39.0) or acute cystitis (ICD-10: N30.0) and having an observation time of at least 365 days prior to the index date, either Canephron^®^ N or Canephron^®^ UNO was prescribed to 3343 (1.40%) and antibiotics to 160,082 (68.74%) on the day of diagnosis. After the exclusion of patients with a combination therapy of Canephron^®^ and antibiotics and patients who received other phytopharmaceuticals as UTI therapy, as well as those taking other UTI drugs (e.g., mannose-, methionine- or arbutin-containing drugs) in the Canephron^®^ cohort, a total of 2320 Canephron^®^ patients and 158,592 antibiotic patients were available for analysis (Figure 1).

Of the 69,450 patients not included in the analysis, as they had been prescribed neither Canephron^®^ nor an antibiotic on the index date, 93% had no documented prescription data for UTI or acute cystitis, while only 7% had received another prescription (3% analgesics, 3% arbutin-containing drugs, and less than 1% other drugs).

A total of 4% of patients in the Canephron^®^ study cohort were also prescribed an analgesic, while in the antibiotic cohort, ~3% received analgesics and just 1% received prescriptions for other drugs (e.g., mannose, methionine, or arbutin-containing drugs).

Table 1 shows the baseline characteristics of the study patients. Canephron^®^ patients were significantly younger (51.3 (SD: 19.9) vs. 55.0 (SD: 20.8) years) and had a slightly lower comorbidity index (1.6 vs. 1.7) than patients who were prescribed antibiotics. The proportion of female patients (81.2 vs. 90.8%) was significantly lower and the proportion of privately insured patients (11.3% vs. 7.6%) significantly higher among patients receiving Canephron^®^ than among the antibiotic cohort. The majority of patients in both cohorts were treated by GPs (90.0% vs. 80.7%). The differences in baseline characteristics indicated the need for age- and sex-stratified analyses.

### 3.2. Sporadic Recurrent Urinary Tract Infection

**Figure 2:**
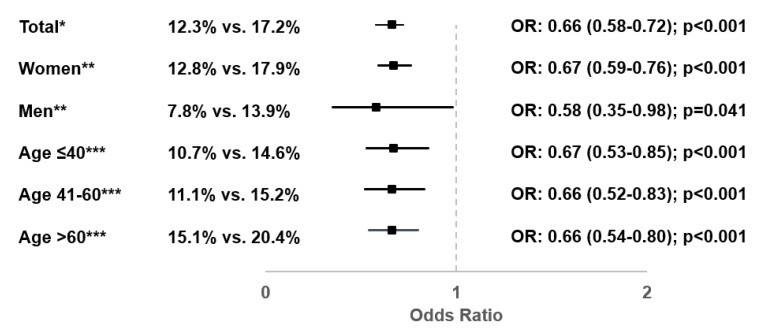
Association between Canephron^®^ prescription and renewed sporadic confirmed diagnosis of UTI within 30–365 days after the index date (Canephron^®^ versus antibiotic). * Multivariable logistic regression adjusted for age, sex, health insurance coverage, practice specialty, and CCI. ** Multivariable logistic regression adjusted for age, health insurance coverage, practice specialty, and CCI. *** Multivariable logistic regression adjusted for sex, health insurance coverage, practice specialty, and CCI.

At least one renewed confirmed diagnosis of UTI was documented within 30–365 days after the index date in 12.3% of patients with Canephron^®^ prescription and 17.2% of patients with antibiotic prescription. Canephron^®^ prescription was associated with significantly lower odds of at least one renewed confirmed diagnosis of UTI within 30–365 days after the index date (odds ratio (OR): 0.66; 95% confidence interval (CI): 0.58–0.72; *p* < 0.001). This association was observed in men and women, and also in different age groups (Figure 2).

### 3.3. Frequent Recurrent Urinary Tract Infections

**Figure 3:**
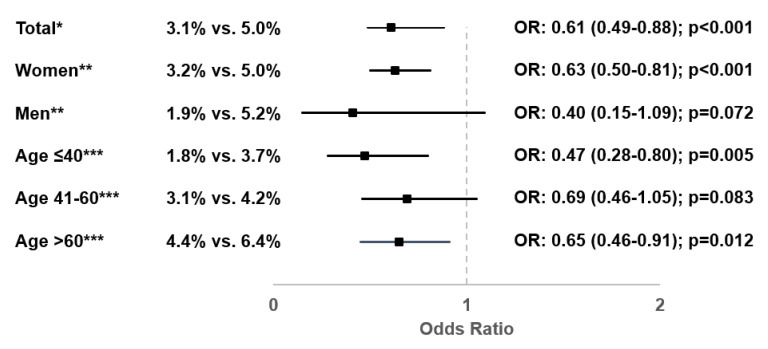
Association between Canephron^®^ prescription and probability of frequent recurrent UTIs (Canephron^®^ versus antibiotic). * Multivariable logistic regression adjusted for age, sex, health insurance coverage, practice specialty, and CCI. ** Multivariable logistic regression adjusted for age, health insurance coverage, practice specialty, and CCI. *** Multivariable logistic regression adjusted for sex, health insurance coverage, practice specialty, and CCI.

At least three diagnoses of UTI 2–365 days after initial diagnosis or at least two diagnoses of UTI 2–184 days after initial diagnosis were documented in 3.1% of patients with Canephron^®^ prescription and 5.0% of patients with antibiotic prescription. Canephron^®^ prescription was associated with significantly lower odds of frequent recurrent UTIs (OR: 0.61; 95% CI: 0.49–0.88; *p* < 0.001). This association was observed significantly or tendentially in men and women, and occurred in all age groups (Figure 3).

### 3.4. Sick Leave Associated with a Urinary Tract Infection

**Figure 4:**
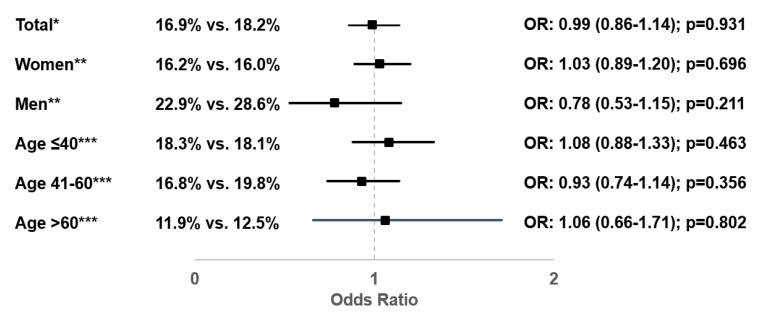
Association between Canephron^®^ prescription and probability of sick leave of at least 3 days (Canephron^®^ versus antibiotic). * Multivariable logistic regression adjusted for age, sex, health insurance coverage, practice specialty, and CCI. ** Multivariable logistic regression adjusted for age, health insurance coverage, practice specialty, and CCI. *** Multivariable logistic regression adjusted for sex, health insurance coverage, practice specialty, and CCI.

In total, 16.9% of patients with Canephron^®^ prescription and 18.2% of patients with antibiotic prescription took at least 3 days of sick leave due to UTIs. There was no significant association between Canephron^®^ prescription and the odds of taking sick leave (OR: 0.99; 95% CI: 0.86–1.14; *p* = 0.931), nor was any such association observed in the subgroups analyzed (Figure 4). A sensitivity analysis was performed to determine the association between Canephron^®^ prescription and the probability of a sick leave of at least 7, 10, or 14 days. No significant associations were observed in the multivariable regression models, and no differences were found between the Canephron^®^ and antibiotic cohorts (OR: 1.01 (95%CI: 0.85–1.19; *p* = 0.949) for ≥7 days: OR: 1.04 (95% CI: 0.86–1.25; *p* = 0.703) for ≥10 days, OR: 1.06 (95% CI: 0.86–1.31; *p* = 0.584) for ≥14 days.

### 3.5. Additional Antibiotic Prescriptions after the Index Date

**Figure 5:**
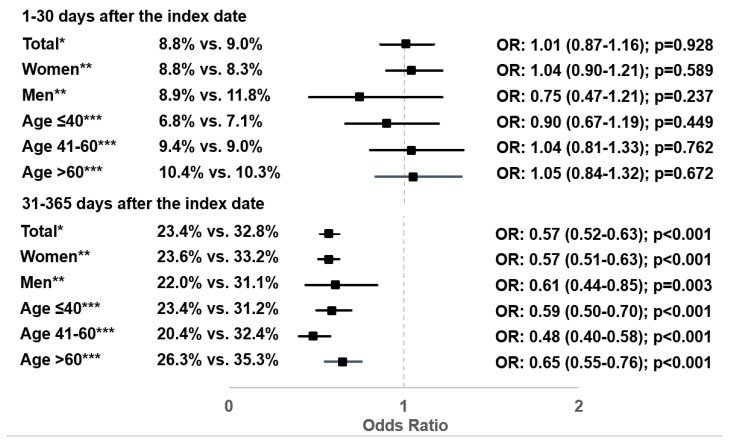
Association between Canephron^®^ prescription and probability of antibiotic prescription 31–365 days or 1–30 days after the index date (Canephron^®^ versus antibiotic). * Multivariable logistic regression adjusted for age, sex, health insurance coverage, practice specialty, and CCI. ** Multivariable logistic regression adjusted for age, health insurance coverage, practice specialty, and CCI. *** Multivariable logistic regression adjusted for sex, health insurance coverage, practice specialty, and CCI.

At least one new (further) antibiotic prescription for UTI was issued 31–365 days after the index date for 23.4% of patients with Canephron^®^ prescription and 32.8% of patients with antibiotic prescription. Canephron^®^ prescription was associated with significantly lower odds of antibiotic prescription 31–365 days after the index date (OR: 0.57; 95% CI: 0.52–0.63; *p* < 0.001). This association was observed in all subgroups investigated (Figure 5). There was, however, no association between Canephron^®^ prescription and antibiotic prescription 1–30 days after the index date (OR: 1.01; 95% CI: 0.87–1.16; *p* = 0.921).

### 3.6. Incidence of Pyelonephritis

Pyelonephritis occurred relatively rarely up to 3 years after the index date. Pyelonephritis was initially documented in 1.6% of Canephron^®^ patients and 1.5% of antibiotic patients, and no significant association was observed in the multivariable Cox regression analysis (Hazard Ratio (HR): 1.00; 95% CI: 0.67–1.48; *p* = 0.954).

## 4. Discussion

To the best of our knowledge, this is the first non-interventional real-world study in Germany proving that Canephron^®^ monotherapy works as a symptomatic treatment for UTI. The data support the important role Canephron^®^ plays in reducing antibiotic use and antibiotic resistance.

The results presented are not surprising, since Canephron^®^ is a well-studied, approved medicinal product. Amdii et al. previously concluded that treatment with Canephron^®^ N allows the use of antibiotics to be reduced in women with uUTIs [25]. Wagenlehner et al. performed a Phase III, non-inferiority trial including 659 women and found a non-inferior difference between patients treated with Canephron^®^ N and those receiving antibiotics, with regard to the proportion of women requiring additional antibiotics [21]. In the present study, two periods (1–30 and 31–365 days after UTI diagnosis) were analyzed for additional antibiotic prescription. There was no significant difference between Canephron^®^ and antibiotic therapy for the period of 1–30 days. This result confirms the outcome of the clinical trial performed by Wagenlehner et al. [21] in a real-world setting. For the period 31–365 days, the use of additional antibiotic prescriptions was significantly lower after Canephron^®^ than after antibiotic therapy.

The prophylactic effect of Canephron^®^ N, used either as combination therapy or monotherapy in patients suffering from UTI, has been reported in several clinical trials [26,27,28,29,30].

A further clinical trial conducted by Davidov and Bunova compared the efficacy and safety of monotherapy with Canephron^®^ N and antibiotic therapy with Ciprofloxacin for the treatment of mild acute cystitis in 160 women [19]. After 6 days of treatment, clinical symptoms had completely disappeared in 66 (82.5%) patients in the Canephron^®^ N group and in 68 (85.0%) patients in the antibiotic group. A relapse of cystitis within one year was observed in 5% of Canephron^®^ N patients and 12.5% of antibiotic patients [19]. Interestingly, RWD show that Canephron^®^ was not only associated with a significantly lower risk of sporadic UTI recurrence but also with a significantly lower risk of frequently recurrent UTIs in all patients.

Canephron^®^ is not only effective for the treatment of UTI, but is also well tolerated [31]. Unlike antibiotics, this herbal medicinal product does not affect the gut microbiota as much as fosfomycin or nitrofurantoin [32]. This is particularly noteworthy, as research into the urinary microbiome has shown that asymptomatic bacteriuria appears to have a protective effect against UTIs [33] and could be considered as a preventive treatment strategy in the case of recurrent infections [34]. The results presented confirm this conclusion, as antibiotic therapy was associated with a significantly higher likelihood of further antibiotic prescriptions in the period of 31–365 days.

In recent years, several non-antibiotic treatment options such as non-steroidal anti-inflammatory drugs (NSAIDs) [8,9,10] and other herbal substances, such as Angocin^®^ [11], have been investigated in order to reduce the number of antibiotic prescriptions. Compared to NSAIDs, Canephron^®^ not only has an analgesic/anti-inflammatory effect but also acts in a variety of ways to alleviate symptoms, due to several components of the drug mixture (1:1:1) of centaury herbs, lovage roots, and rosemary leaves. Thanks to its spasmolytic [14], anti-inflammatory, and anti-nociceptive [18] effects, Canephron^®^ has the potential to reduce symptoms associated with acute lower uUTIs, such as inflammatory pain, spasm, and frequent micturition. In addition, the anti-adhesive effects [17] support the elimination of bacteria from the urinary tract. The present retrospective study showed that there is acceptance of non-antibiotic treatment options, as almost one third of the patients (31.3%) in clinical practice received no antibiotic therapy. Over 2320 of these cases were treated with Canephron^®^ monotherapy. GPs prescribed Canephron^®^ more often than gynecologists or urologists, reflecting the tendency of patients in Germany suffering from a uUTI to first consult a GP.

Pyelonephritis can occur as complication in 0.3–0.5% of lower urinary tract infections [8,10]. Although higher rates of pyelonephritis have been reported in patients treated with alternative treatment options than those treated with antibiotics [8,10,21], a retrospective long-term follow-up analysis showed that non-antibiotic treatment has no negative impact on pyelonephritis [9]. The present study also found no significant association between Canephron^®^ and the incidence of pyelonephritis. In addition, the duration of UTI-related sick leave under Canephron^®^ therapy was as long as that under antibiotic treatment.

These results support the hypothesis that symptomatic therapy with Canephron^®^ should be used preferentially for uUTIs instead of “a priori” antibiotic treatment.

The strengths of this study include the large dataset (over 2300 patients receiving Canephron^®^ and more than 158,000 antibiotic patients), which includes both female and male patients, as well as different age groups and physician specialties. Furthermore, our study used the German IMS^®^ DA database, the reliability of which has been validated in several medical studies, and consequently supports the reliability of our findings [22,35].

The IMS^®^DA database used exhibits specific characteristics which have to be considered. First, assessments rely on ICD codes entered by GPs, urologists, or gynecologists. These codes are checked and validated by the owner of the IMS^®^DA database (IQVIA). Nevertheless, ICD codes do not allow for differentiation between either complicated and uncomplicated diagnoses or UTI severities. Second, the number of patients treated with Canephron^®^ was markedly lower than the number of patients treated with antibiotics. The lower number of prescriptions for the herbal medicinal product may be due to its regulatory status in Germany, which is over the counter (OTC), with no prescription required, whereas antibiotics are prescription only (Rx). The database, however, only includes data on the use of herbal medicines that patients have been prescribed by their physicians. The same applies to NSAIDs. It is possible that physicians decided not to prescribe any medication to patients with very mild UTIs or just gave them recommendations for OTC products that patients could have bought in the pharmacies afterwards by themselves. In addition, no data were available on socioeconomic status and lifestyle-related risk factors (smoking, alcohol, physical activity). In addition, patients can only be observed in a single practice; if they receive a diagnosis or prescription from another physician, such prescriptions are not documented.

To reduce any potential bias, regression models were applied separately for three age groups and for men and women, in addition to the adjustment included in the regression models.

In conclusion, this study revealed that Canephron^®^ is already being used as monotherapy for treating UTI in a real-world setting. In addition, despite the limitations due to the database used, the results demonstrate that symptomatic treatment with Canephron^®^ as a monotherapy is successful and safe. The rates of sporadic and frequent UTI recurrences were even significantly lower with Canephron^®^ than with antibiotics. Therefore, symptomatic treatment of uncomplicated lower UTI with Canephron^®^, an herbal medicinal product, is recommended to reduce prescriptions of antibiotics for this indication.

## 5. Conclusions

These real-world data show that Canephron^®^ is an effective, safe symptomatic treatment for acute cystitis or UTI. Long-term additional antibiotic prescriptions and UTI recurrences were also significantly lower after Canephron^®^ therapy than after antibiotic therapy. Therefore, Canephron^®^ should be considered as an alternative treatment, particularly to also strengthen antimicrobial stewardship strategies.

## Figures and Tables

**Table 1:** Basic characteristics of study patients.

Variable	Patients with Canephron^®^ Prescription	Patients with Antibiotic Prescription	*p*-Value
N	2320	158,592	
Age (mean, SD)	51.3 (19.9)	55.0 (20.8)	<0.001
≤40 years (%)	33.2	29.5	<0.001
41–60 years (%)	32.3	28.6	
>60 years (%)	34.5	41.9	
Sex: female (%)	81.2	90.8	<0.001
CCI (mean, SD)	1.6 (2.3)	1.7 (2.4)	0.019
Private health insurance coverage (%)	11.3	7.6	<0.001
Therapy by general practitioners	90.0	80.7	<0.001
Therapy by gynecologists	7.1	14.1	
Therapy by urologists	2.9	5.2	

## Data Availability

The data presented in this study are available on request from the corresponding author.
